# Body Composition: Assessment, Regulation, and Emerging Techniques

**DOI:** 10.1155/2013/125068

**Published:** 2013-06-13

**Authors:** Analiza M. Silva, David A. Fields, Diana Thomas, Boyd J. Strauss

**Affiliations:** ^1^Exercise and Health Laboratory, CIPER, Fac Motricidade Humana, Univ Tecn Lisboa, 1499-002 Cruz Quebrada, Portugal; ^2^Department of Pediatrics, CMRI Metabolic Research Program, University of Oklahoma Health Sciences Center, Oklahoma City, OK 73104, USA; ^3^Center for Quantitative Obesity Research, Montclair State University, Montclair, NJ 07043, USA; ^4^Department of Medicine, Southern Clinical School, Monash University, Melbourne, VIC 3168, Australia

Advances in body composition measurement, evaluation, and analysis have contributed vastly to our knowledge of human biology. These advances can be organized into three distinct, interconnected areas: body composition paradigms, body composition methodology, and body composition response to external influences. The first research area describes the architecture of human body composition identifying proportions of various compartments and their steady-state associations among the atomic, molecular, cellular, tissue system, and whole-body levels; the second research area investigates the merits of different body composition measurement methods for *in vivo*; and the third investigates the response of body composition to factors such as growth and aging.

The five-level model proposed by Z. Wang et al. in 1992 was an important advancement towards building an appropriate structure for body composition research by organizing components into five distinct levels of increasing complexity: atomic, molecular, cellular, tissue system, and whole-body.

The eight articles that appear in this special issue are categorized with some overlap into these three main research areas. The review paper proposed by A. M. Silva et al. provides the relationship between body composition architecture and methodology research. The authors discuss the assumptions of hydrometric and densitometric two-component models, namely, 73.2% and 1.1 g/cc for the fat-free mass (FFM) hydration and density, correspondingly. These averages do not include interindividual variability in FFM, particularly in the pediatric population. The review highlights the need for multicomponent models for more accurate body composition assessment in children. To meet this need, the authors reviewed skinfolds and bioelectrical impedance-based predictive equations developed using multicomponent models for use as a reference method.

In general, development of methodology, methodology evaluation, and methodology validation was the main focus of one review and three original papers. These articles develop mathematical formulae derived from statistical analysis of experimental observations. At the whole-body level of analysis, A. F. Casey reviewed assessment validity and reliability in individuals with intellectual disability. D. Machado et al. proposed new anthropometric-based equations to assess bone mineral, lean soft tissue, and fat mass for a male pediatric population. E. Forsum et al. analyzed the relationship between body mass index and body fat assessed by air displacement plethysmography in 4-year-old children. At the tissue level of analysis, W. Shen et al. compared bone marrow fat measurements among T1-weighed magnetic resonance imaging (MRI) and magnetic resonance spectroscopy. E. L. Rolfe et al. analyzed the validity of ultrasound visceral and subcutaneous abdominal depth as a proxy for MRI measured internal abdominal and subcutaneous fat in infants.

Finally, in the last research category, two original papers investigated body composition response to aging and ethnicity. M. F. Almeida et al. examined longitudinal anthropometric changes in Brazilian older adults from 2000 to 2006. C. L. Carpenter et al. evaluated the efficacy of the body mass index (BMI) as a proxy for adiposity across different ethnic groups by comparing to measured percent body fat. The research field investigating body composition responses also includes responses to growth, development, nutrition, exercise, hormonal changes, and medication. In this regard, the study of the dynamic relations between body components and its associate's functions has been recently explored. A large body of research evaluating body composition responses to these factors at both the experimental and theoretical levels is now available. In fact, the concept of functional body composition has been proposed, providing more sophisticated view of nutritional status, metabolism, endocrinology, and diseases. More than just the assessment of the compartments in any of these five levels, functional body composition addresses the quantitative and biological interactions of these compartments with energy balance status, metabolic features, healthy and unhealthy biomarkers, sports performance markers, and many other measurable physiological expressions of a living organism.

In conclusion, the original research and reviews contained in this special issue identify gaps and provide potential alternatives to more accurate routine body composition assessment in basic science and clinical settings. From a clinician's perspective, these papers mostly tell us about body composition models, techniques, and assessment in health across the age span, with the underlying assumption of steady state. However, these papers do not address how robust these techniques are in a nonsteady state (i.e. clinical illness). This is important in an obesodiabetogenic environment that appears to hit all corners of the world. This aspect is also intimately linked to the functional aspects of body composition where a key question is the nature and the extent of pathophysiological influences on body composition changes, on the one hand, and the effect of those body composition changes on pathophysiology on the other hand. What these papers do is to help in setting a modern scene for interpreting clinical findings, though more research is needed to capture the longitudinal body composition response and related energy regulation during clinical illness, growth, and aging, as well as the effect of diet and exercise interventions.

*Analiza M. Silva* *Analiza M. Silva* *David A. Fields* *David A. Fields* *Diana Thomas* *Diana Thomas* *Boyd J. Strauss* *Boyd J. Strauss*

